# Asthma and COPD management of patients with intellectual disabilities in general practice

**DOI:** 10.1038/s41533-024-00375-w

**Published:** 2024-06-26

**Authors:** Mathilde Mastebroek, Nadeem C. M. Everlo, Maarten Cuypers, Erik W. M. A. Bischoff, Bianca W. M. Schalk

**Affiliations:** Department of Primary and Community Care, Research Institute for Medical Innovation, Radboud University Medical Center, Nijmegen, The Netherlands

**Keywords:** Health services, Outcomes research, Physical examination

## Abstract

People with intellectual disabilities experience overall poorer health and healthcare access than the general population. It is largely unknown how this applies to asthma and chronic obstructive pulmonary disease (COPD) management by general practitioners (GPs). In a 10-year retrospective matched cohort study, n = 34,429, we examined year prevalence of asthma and COPD in adult patients with and without intellectual disabilities and potential differences in the delivery of asthma and COPD disease management activities in Dutch general practices (2010–2019). We collected information on patient characteristics, comorbidity, consultation patterns, use and outcomes of asthma/COPD control questionnaires, spirometry measurement, pulmonology referrals, and prescribed medication. Asthma patients with intellectual disabilities suffered more frequently from obesity (53.2% vs. 39.5% without intellectual disabilities), and both asthma and COPD patients with intellectual disabilities were more frequently current smokers (45.2% vs. 22.1% without intellectual disabilities, and 76.6% vs. 51.4% without intellectual disabilities, respectively). Also, a statistically significant larger number of asthma patients with intellectual disabilities were prescribed antibiotics (69.9% vs. 54.5%). COPD patients with intellectual disabilities, compared with matched controls without intellectual disabilities, received significantly more often either no COPD-related practice consultation at all (respectively 20.8% vs. 8.5%, p = 0.004) or a large number of practice consultations (>31 consultations, respectively 16.7% vs. 5.3%, p = 0.004). For asthma, there was no statistical difference between patients with or without intellectual disabilities regarding the number and type of consultations. The asthma year point prevalence in patients with intellectual disabilities was, from 2014 onward, significantly higher, and in 2019 was 8.7% vs. 6.0% for people without intellectual disabilities. For COPD, it was comparable in both groups. Both asthma and COPD patients with intellectual disabilities appeared considerably younger in age than patients without intellectual disabilities. Our findings warrant further research into the causes of the differences found for asthma and COPD and whether they also infer differences in the quality or the effectiveness of GP disease management, especially for young adults with intellectual disabilities.

## Introduction

People with intellectual disabilities, characterised by significant limitations in both intellectual functioning and adaptive behaviour with an onset in the developmental period^1^, have poorer health than the general population. They experience higher rates of chronic conditions and multimorbidity, a shorter life expectancy, and increased risk of early death^2–6^. These rates apply also to pulmonary conditions. Research on asthma and chronic obstructive pulmonary disease (COPD) in people with intellectual disabilities indicates that adequate management of these conditions by general practitioners (GPs) is challenging for this patient group, resulting in poor health outcomes^6–10^. Learning difficulties, including intellectual disabilities, as a psychosocial factor, are associated with a threefold increase in risk of death from asthma^7^. Decedents with intellectual disabilities, compared with a matched cohort, had in the last year of life increased odds of presentation at an emergency department, of hospitalisation, and of death from respiratory disorders (odds ratio 2.6, 2.5, and 1.9, respectively)^8^. For asthma specifically, they were around five times more likely to present at an emergency department or have a hospital admission for asthma (relative risk 4.7 and 4.6, respectively)^8^. Lastly, people with mild intellectual disabilities and a respiratory disorder are around five times as likely as controls without intellectual disabilities to have poor lung function measures^6^. Asthma also appears more prevalent in people with intellectual disabilities, with prevalence numbers varying between 4.7% and 21.1% and a 1.3%- to 6.0%-point excess compared with people without intellectual disabilities^2,6,11–17^, although some studies have found no significant difference^14,18^. The prevalence of COPD in people with intellectual disabilities is less studied and is supposedly comparable to the general population or lower in people with intellectual disabilities^19^.

Asthma and COPD are diseases with both a high comorbidity and a significant impact on quality of life, which will be exacerbated if the GP care for these conditions is not coordinated adequately^20,21^. Poor management of these conditions may relate to characteristics of the patient group itself, such as difficulties in communication and health awareness, but also to factors concerning the organisation and provision of asthma and COPD care by GPs, such as ill-suited instruments or lack of adjustments in communication, treatment, and screening^22,23^. Specific information on how asthma and COPD in patients with intellectual disabilities are managed in GP care is scarce. A study focusing on potential differences in care delivery and treatment-related patient characteristics would be helpful in identifying differences in management. Consequently, it may offer explanations for the poor health outcomes for these conditions in people with intellectual disabilities and clues for GPs as to the elements of treatment and monitoring that require additional attention in practice. We have therefore performed a 10-year retrospective matched cohort study to examine (1) prevalence rates for each cohort year and (2) potential differences in the delivery of primary care disease management for asthma and COPD to adult patients with intellectual disabilities in the first year of diagnosis and onward compared with the general population.

## Methods

Data were derived from the general practitioner database of the Department of Primary and Community Care at the Radboud University Medical Centre in the Netherlands, covering about 100 general practices with more than 450,000 patients. For this retrospective matched cohort study, we used dynamic cohort data from 79 of these practices, covering the study period 2010–2019.

For research purposes, the general practices cooperating with the Department of Primary and Community Care follow additional training and regulations regarding ICPC coding in patient records and health information systems, entailing mandatory registration of an ICPC code in each consultation. In addition, enrolment of patients in funded disease management programmes for asthma and COPD offers an incentive to GPs for ICPC coding.

All enroled patients and legal representatives have been informed about the standardised use of their encoded data for research purposes and may object to its use at any time. The Research Ethics Committee (CMO Region Arnhem – Nijmegen) waived the need for ethical approvement. We report in accordance with the Strengthening the Reporting of Observational Studies in Epidemiology (STROBE) statement^24^.

### Selection and matching procedure

**Fig. 1:**
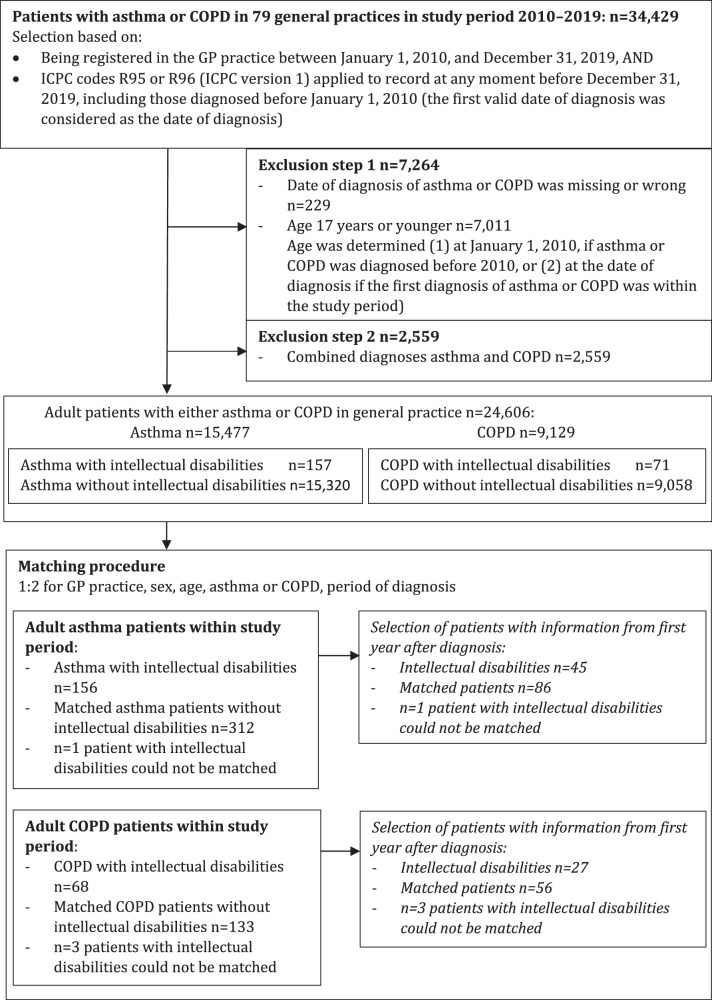
Flowchart of the selection and matching of the study population. First step: selection of patients with either asthma or COPD. Second step: identification of patients with intellectual disabilities within the groups of patients with asthma or COPD. Third step: matching of patients with intellectual disabilities and asthma or COPD with asthma and COPD patients without intellectual disabilities. Fourth step: selection of patients with medical file information from the first year after diagnosis. GP General Practitioner, ICPC International Classification of Primary Care.

In our cohort, we included all adult patients with either COPD or asthma (n = 34,429, based on ICPC codes R95 or R96, respectively), following the selection procedure displayed in Fig. 1. To identify patients with intellectual disabilities in the total database, we followed a procedure encompassing a selection of diagnostic ICPC codes frequently used to record the presence of an intellectual disabilities, in combination with intellectual disability-related text entries (described in Supplementary Methods).

Application of the selected exclusion criteria (patients younger than 18 years and patients with combined diagnoses of asthma and COPD) resulted in the inclusion of 15,320 adult asthma patients without intellectual disabilities and 157 with intellectual disabilities, and 9058 COPD patients without intellectual disabilities and 71 with intellectual disabilities (Fig. 1). From the cohort of asthma and COPD patients without intellectual disabilities, a matched study group (controls) was created in a 1:2 ratio for GP practice, sex, age subgroup (age 18–34/35–49/50–64/65+ years), diagnosis of either asthma or COPD, and diagnosis before or after January 1, 2010 (Fig. 1). We chose 1:2 matching and these specific variables to avoid confounding and ensure comparability between patients, while maintaining precision in estimating the effect of the presence of intellectual disabilities on delivery of care.

### Patient characteristics and primary care disease management activities

From the GP database, we collected data regarding patients’ years of follow-up during the study period, smoking status, presence and types of comorbidity generally often co-occurring with asthma and COPD, including obesity^25^. Follow-up was defined as the number of years between (1) the date of diagnosis, or, if the diagnosis was made before 1 January 2010, the start date of the study or date of enrolment in the GP practice, and (2) the end date of the study or the date on which the patient was no longer registered in the GP practice. Primary care disease management activities during the years of follow-up included consultation patterns, spirometry measurements, pulmonology referrals, medication prescriptions related to the management of asthma or COPD, and the application and results of the Asthma Control Questionnaire (ACQ)^26^ or the Clinical COPD Questionnaire (CCQ)^27^, the Medical Research Council dyspnoea scale (MRC)^28^, and the GOLD COPD severity grading (Global Initiative for Chronic Obstructive Lung Disease)^29^. Respiratory medication was identified using the Anatomical Therapeutic Classification (ATC) codes in the RO3 category and for systemic antibiotics and systemic prednisone/prednisolone^30^. See Supplementary Methods for further details of the operationalisation of all used parameters.

### Statistical analysis

Analyses were performed using the statistical package SPSS 25. The year point prevalence of asthma and COPD was calculated by dividing the number of adult cases of asthma or COPD by the total number of persons in the GP practices in the particular year of interest. This was done separately for adults with and without intellectual disabilities. Per year, we performed a chi-square test using a two-by-two table of cases (Asthma or COPD: yes/no) vs. adults (intellectual disabilities: yes/no). The delivered primary care disease management for asthma and COPD was analysed as the total number of activities generated for the total number of patients with intellectual disabilities and the controls during the full study period after diagnosis and, for the first year after diagnosis, separately if applicable during the study period. If the distribution of follow-up years was comparable between matched groups with and without intellectual disabilities, then no calculation was made for delivered primary care per study year. The frequency rates of care activities were compared between persons with intellectual disabilities and their controls using numbers and percentages and were tested with the chi-square test. Continuous variables for delivered care were expressed as median (25th percentile–75th percentile: p25–p75), and differences between persons with and without intellectual disabilities were tested with the Mann-Whitney test. In all analyses, statistical significance was set at a two-sided p value (p) ≤ 0.05.

## Results

### Prevalence of asthma and COPD

In the 10-year period studied, the year point prevalence of asthma rose in each subsequent study year: for patients without intellectual disabilities from 4.2% in 2010 to 6.0% in 2019, whereas in patients with intellectual disabilities, it increased from 4.1% in 2010 to 8.7% in 2019 (Table 1). From 2014 onwards, the asthma point prevalence in patients with intellectual disabilities was significantly higher than in the group without intellectual disabilities. Regarding COPD, the year point prevalence rose slightly in patients with intellectual disabilities, climbing from 1.2% in 2010 to 2.3% in 2019, and was significantly lower than in patients without intellectual disabilities from 2010 to 2016. From 2017 onwards, the COPD point prevalence of patients with and without intellectual disabilities was comparable and around 2.4% in 2019.

**Table 1:** Number of patients and prevalence of asthma and COPD per study year.

Study year	Persons asthma and intellectual disabilities *n* (prevalence in %)	Persons asthma, no intellectual disabilities *n* (prevalence in %)	Difference prevalence asthma per year, %-point	Persons COPD and intellectual disabilities *n* (prevalence in %)	Persons COPD, no intellectual disabilities *n* (prevalence in %)	Difference prevalence COPD per year, %-point
2010	87 (4.1%)	2119 (4.2%)		26 (1.2%)**	4366 (2.1%)	−0.84
2011	97 (4.5%)	2180 (4.3%)		31 (1.4%)*	4747 (2.2%)	−0.74
2012	110 (4.9%)	2264 (4.7%)		36 (1.6%)*	5210 (2.3%)	−0.74
2013	123 (5.3%)	2310 (4.8%)		39 (1.7%)*	5584 (2.4%)	−0.66
2014	146 (6.3%)**	2333 (5.0%)	+1.25	40 (1.7%)*	5865 (2.4%)	−0.70
2015	157 (6.7%)**	2355 (5.3%)	+1.42	41 (1.7%)*	6131 (2.5%)	−0.71
2016	172 (7.2%)***	2391 (5.5%)	+1.72	42 (1.8%)*	6315 (2.4%)	−0.68
2017	189 (7.8%)***	2431 (5.7%)	+2.12	48 (2.0%)	6484 (2.4%)	
2018	205 (8.3%)***	2463 (5.9%)	+2.47	52 (2.1%)	6629 (2.4%)	
2019	217 (8.7%)***	2485 (6.0%)	+2.74	56 (2.3%)	6863 (2.4%)	

^*^p ≤ 0.05, **p ≤ 0.01, ***p ≤ 0.001

### Population characteristics

After matching, we included 156 adult asthma patients with intellectual disabilities and 312 without intellectual disabilities, and 68 adult COPD patients with intellectual disabilities and 133 without intellectual disabilities (Fig. 1 and Table 2). Matching was successful for the selected matching variables, and both groups also appeared comparable with regard to follow-up: follow-up data for at least five years were present for approximately 65% of asthma patients and 53% of COPD patients, either with or without intellectual disabilities. However, before matching, in both the intellectual disabilities groups with asthma or COPD, the two youngest age groups (age 18–34 and 35–49) were significantly larger compared with patients without intellectual disabilities (p ≤ 0.001, Table 2). In matched adults with intellectual disabilities and either asthma or COPD, current smoking rates were significantly higher compared with adults without intellectual disabilities: 45.2% (38/84) vs. 22.1% (32/145), respectively, for asthma (p = 0.001) and 76.6% (36/47) vs. 51.4% (54/105), respectively, for COPD (p = 0.014) (Table 2). Significantly more asthma patients with intellectual disabilities were obese compared with their matched peers without intellectual disabilities: 53.2% vs. 39.5% (p = 0.03). They also suffered significantly more frequently from diabetes (14.7% vs. 8.3%, p = 0.03) and a diagnosis of anxiety and/or depression (34.5% vs. 21.8%, p = 0.003).

**Table 2:** Population characteristics unmatched and matched study group.

Characteristics unmatched study group	Persons intellectual disabilities and asthma (*n* = 157) n (%)	Persons asthma no intellectual disabilities (*n* = 15,319) n (%)	Persons intellectual disabilities and COPD (*n* = 71) n (%)	Persons COPD no intellectual disabilities (*n* = 9058) n (%)
Sex				
Male	69 (43.9%)	6394 (41.7%)	35 (49.3%)	4997 (55.2%)
Female Unknown	88 (56.1%)	8924 (58.3%) 1 (0.0%)	36 (50.7%)	4061 (44.8%)
Age (years)	Statistical significance*** (comparison patients with and without intellectual disabilities in each age group)		Statistical significance*** (comparison patients with and without intellectual disabilities in each age group)	
18–34	80 (51.0%)	5139 (33.5%)	2 (2.8%)	50 (0.6%)
35–49	53 (33.8%)	4719 (30.8%)	19 (26.8%)	892 (9.8%)
50–64	20 (12.7%)	3561 (23.2%)	36 (50.7%)	3714 (41.0%)
≥65	4 (2.5%)	1900 (12.4%)	14 (19.7%)	4402 (48.6%)

Characteristics matched study group	Persons intellectual disabilities and asthma (n = 156) n (%)	Matched controls asthma no intellectual disabilities (n = 312) n (%)	Persons intellectual disabilities and COPD (n = 68) n (%)	Matched controls COPD no intellectual disabilities (n = 133) n (%)
Sex				
Male	69 (44.2%)	138 (44.2%)	34 (50.0%)	66 (49.6%)
Female	87 (55.8%)	174 (55.8%)	34 (50.0%)	67 (50.4%)
Age (years)				
18–34	80 (51.3%)	160 (51.3%)	0	0
35–49	52 (33.3%)	104 (33.3%)	18 (26.5%)	33 (24.8%)
50–64	20 (12.8%)	40 (12.8%)	36 (52.9%)	72 (54.1%)
≥65	4 (2.6%)	8 (2.6%)	14 (20.6%)	28 (21.1%)
Smoking status	Statistical significance*** (comparison patients with and without intellectual disabilities in each category)		Statistical significance*** (comparison patients with and without intellectual disabilities in each category)	
Yes	38 (45.2%)	32 (22.1%)	36 (76.6%)	54 (51.4%)
Never	25 (29.8%)	71 (49.0%)	2 (4.3%)	8 (7.6%)
Stopped	21 (25.0%)	42 (29.0%)	9 (19.1%)	43 (41.0%)
Missing information	72	167	21	28
Year of diagnosis				
<2010	93 (59.6%)	186 (59.6%)	26 (38.2%)	50 (37.6%)
2010–2019	63 (40.4%)	126 (40.4%)	42 (61.8%)	83 (62.4%)
Years of follow-up during the study period				
<1 year	10 (6.4%)	18 (5.8%)	8 (11.8%)	19 (14.3%)
1–4 years	46 (29.5%)	86 (27.6%)	23 (33.8%)	45 (33.8%)
5–9 years	34 (21.8%)	66 (21.2%)	24 (35.3%)	39 (29.3%)
10 years	66 (42.3%)	142 (45.5%)	13 (19.1%)	30 (22.6%)
Comorbidity				
Obesity	58 (53.2%)*	64 (39.5%)*	17 (32.7%)	26 (23.6%)
Missing information	47	150	16	23
Hypertension	27 (17,3%)	44 (14.1%)	23 (33.8%)	58 (43.6%)
Ischaemic heart disease	7 (4.5%)	18 (5.8%)	8 (11.8%)	25 (18.8%)
Heart failure	2 (1.3%)	4 (1.3%)	4 (5.9%)	17 (12.8%)
Cerebrovascular accident	3 (1.9%)	4 (1.3%)	6 (8.8%)	9 (6.8%)
Diabetes	23 (14.7%)*	26 (8.3%)*	12 (17.6%)	20 (15.0%)
Anxiety/depression	54 (34.6%)**	68 (21.8%)**	26 (38.2%)	36 (27.1%)
Osteoporosis	3 (1.9%)	6 (1.9%)	5 (7.4%)	17 (12.8%)
Osteoarthritis	11 (7.1%)	24 (7.7%)	12 (17.5%)	28 (21.1%)

^*^p ≤ 0.05, **p ≤ 0.01, ***p ≤ 0.001.

### Delivered care for asthma and COPD

In the majority of included general practices (71%), it was possible to distinguish the number and type of consultations exclusively for asthma or COPD. The health information systems in the remaining general practices did not allow anonymised extraction of consultation dates combined with ICPC codes. For asthma, there was no statistical difference between patients with or without intellectual disabilities regarding the number and type of consultations. For COPD, in the study period, patients with intellectual disabilities compared with matched controls received statistically significantly more often either no COPD-related practice consultation at all (respectively 20.8% vs. 8.5%, p = 0.004) or a large number of practice consultations (>31 consultations, respectively 16.7% vs. 5.3%, p = 0.004) (Table 3). We saw no statistically significant differences between COPD patients with and without intellectual disabilities for the other types of consultations, nor in the first year of diagnosis.

**Table 3:** Care contacts for adults related to asthma or COPD within the overall study period and within the first year after diagnosis.

Type of care contacts related to asthma	Asthma - Overall study period		Asthma - In 1st year after diagnosis		Type of care contacts related to COPD	COPD - Overall study period		COPD - In 1st year after diagnosis	
	Persons intellectual disabilities and asthma (*n* = 107)	Matched controls asthma no intellectual disabilities (*n* = 214)	Persons intellectual disabilities and asthma (*n* = 30)	Matched controls asthma no intellectual disabilities (*n* = 54)		Persons intellectual disabilities and COPD (*n* = 48)	Matched controls COPD no intellectual disabilities (*n* = 94)	Persons intellectual disabilities and COPD (*n* = 15)	Matched controls COPD no intellectual disabilities (*n* = 37)
Total number with ≥1 care contact of any category *n* (%)	82 (52.6%)	155 (49.7%)	27 (90.0%)	49 (90.7%)	Total number with ≥1 care contact of any category *n (%)*	42 (61.8%)	89 (66.9%)	14 (93.3%)	37 (100%)
No contact	25 (23.4%)	59 (27.6%)			No contact	6 (12.5%)	5 (5.3%)		
1–2 times	22 (20.6%)	52 (24.3%)			1–10 times	19 (39.6%)	35 (37.2%)		
3-10 times	36 (33.6%)	68 (31.8%)			11–30 times	14 (29.2%)	43 (45.7%)		
11+ times	24 (22.4%)	35 (16.4%)			31+ times	9 (18.8%)	11 (11.7%)		
Median (p25–p75)	3 (1–9)	2 (0–7.3)	3 (1–7.3)	3 (1–8)	Median (p25–p75)	8 (2–23.4)	12 (4–22)	14 (0–11)	11 (4–21.5)
Care contacts subcategories									
Practice consultations									
Total number with ≥1 care contact *n (%)*	80 (51.3%)	141 (45.2%)	27 (90.0%)	43 (79.6%)	Total number with ≥1 care contact *n (%)*	38 (55.9%)	86 (64.7%)	13 (86.7%)	35 (94.6%)
No contact	27 (25.2%)	73 (34.1%)			No contact	10 (20.8%)**	8 (8.5%)**		
1–2 times	31 (29.0%)	49 (22.9%)			1–10 times	23 (47.9%)**	47 (50.0%)**		
3–10 times	32 (29.9%)	69 (32.2%)			11–30 times	7 (14.6%)**	34 (36.2%)**		
11+ times	17 (15.9%)	23 (10.7%)			31+ times	8 (16.7%)**	5 (5.3%)**		
Median (p25–p75)	2 (0–6)	1 (0–5)	2 (1–4)	3 (1–6.3)	Median (p25–p75)	5 (1–20.3)	8.5 (3–15)	6 (2–23)	8 (3.5–19)
Telephone consultations									
Total number with ≥1 care contact *n (%)*	51 (32.7%)	89 (28.5%)	12 (40.0%)	31 (57.4%)	Total number with ≥1 care contact *n (%)*	33 (48.5%)	66 (49.6%)	14 (93.3%)	25 (67.6%)
No contact	56 (52.3%)	125 (58.4%)			No contact	15 (31.3%)	28 (29.8%)		
1–2 times	24 (22.4%)	56 (26.2%)			1–2 times	14 (29.2%)	29 (30.9%)		
3–4 times	13 (12.1%)	16 (7.5%)			3–4 times	7 (14.6%)	17 (18.1%)		
5+ times	14 (13.1%)	17 (7.9%)			5+ times	12 (25.0%)	20 (21.3%)		
Median (p25–p75)	0 (0–3)	0 (0-1)	0 (0–2.3)	1 (0-2)	Median (p25–p75)	1.5 (0–4.8)	2 (0–4)	2 (1–5)	1 (0–4)
Home visits during office hours									
Total number with ≥1 care contact *n (%)*	3 (1.9%)	9 (2.9%)	1 (3.3%)	2 (3.7%)	Total number with ≥1 care contact *n (%)*	18 (26.5%)	24 (18.0%)	4 (26.7%)	4 (10.8%)
No contact					No contact	30 (62.5%)	70 (74.5%)		
1–2 time					1–2 time	10 (20.8%)	10 (10.6%)		
3+ times					3+ times	8 (16.7%)	14 (14.9%)		
Median (p25–p75)	NA	NA	NA	NA	Median (p25–p75)	0 (0–1)	0 (0–1)	0 (0–1)	0 (0–0)
Out-of-hours contacts									
Total number with ≥1 care contact *n (%)*	5 (3.2%)	1 (0.3%)			Total number with ≥1 care contact *n* (%)	4 (5.9%)	6 (4.5%)	0	3 (8.1%)
1 time					1 time				
2–3 times					2–3 times				
>3 times					>3 times				
Median (p25–p75)	NA	NA			Median (p25–p75)	NA	NA	NA	NA

*NA* not applicable.

^*^ *p* ≤ 0.05, ** *p* ≤ 0.01, *** *p* ≤ 0.001.

During the study period, the percentage of asthma patients having received a spirometry measurement, an Asthma Control Questionnaire, or a pulmonology referral did not differ statistically significantly between the two groups, nor in the first year of diagnosis (Table 4). In addition, we found no differences in the qualitative results of the Asthma Control Questionnaire between patients with or without intellectual disabilities. For COPD, the same pattern of no statistically significant differences between patients with and without intellectual disabilities applied regarding spirometry, application and results of questionnaires, and pulmonology referrals (Table 4).

**Table 4:** Care activities for adults with asthma or COPD within the overall study period and within the first year after diagnosis.

Type of care activity	Asthma - Overall study period		Asthma - In 1st year after diagnosis		COPD - Overall study period		COPD - In 1st year after diagnosis	
	Persons intellectual disabilities and asthma (*n* = 156)	Matched controls asthma no intellectual disabilities (*n* = 312)	Persons intellectual disabilities and asthma (*n* = 45)	Matched controls asthma no intellectual disabilities (*n* = 86)	Persons intellectual disabilities and COPD (*n* = 68)	Matched controls COPD no intellectual disabilities (*n* = 133)	Persons intellectual disabilities and COPD (*n* = 27)	Matched controls COPD no intellectual disabilities (*n* = 56)
Spirometry assessment n (%)^a^	60 (38.5%)	113 (36.2%)	9 (20.0%)	31 (36.0%)	34 (50.0%)	80 (60.2%)	8 (29.6%)	26 (46.4%)
*Questionnaire*								
ACQ n (%)^a^	23 (14.7%)	48 (15.4%)	1 (2.2%)	5 (5.8%)				
ACQ < 0.75 ‘good control’ n (%)	12 (52.2%)	25 (54.3%) (missing n = 2)	1 (2.2%)	2 (40.0%)				
CCQ n (%)^a^					26 (38.2%)	67 (50.4%)	4 (38.2%)	15 (50.4%)
Median score (p25–p75)					1.9 (1.5–2.8)	1.5 (1–2.4)	2.5 (1.8–3.3)	1.8 (0.8–3.3)
MRC Dyspnoea Scale n (%)^a^					29 (42.6%)	73 (54.9%)	4 (42.6%)	15 (54.9%)
Grades 0–1					14 (50.0%)	40 (57.1%)	0	9 (60.0%)
Grades 2–3					9 (32.1%)	25 (35.7%)	4 (100%)	6 (40.0%)
Grades 4–5					5 (17.9%)	5 (7.1%)	0	0
GOLD stage								
1 mild					12 (41.4%)	28 (40.0%)	2 (33.3%)	6 (35.3%)
2 moderate					10 (34.5%)	35 (50.0%)	4 (66.7%)	11 (64.7%)
3–4 (very) severe					7 (24.1%)	7 (10.0%)	0	0
Referrals to pulmonologist n (%)^a^	13 (8.3%)	19 (6.1%)	2 (4.4%)	2 (2.3%)	6 (8.8%)	18 (13.5%)	2 (7.4%)	0
Medication								
Total^b^ n (%)^a^	121 (77.6%)	244 (78.2%)	30 (66.7%)	67 (77.9%)	54 (79.4%)	106 (79.7%)	17 (63.0%)	38 (67.9%)
Predniso(lo)ne n (%)^a^	50 (32.1%)	82 (26.3%)	5 (11.1%)	6 (7.0%)	33 (48.5%)	66 (49.6%)	3 (11.1%)	7 (12.5%)
Antibiotics n (%)^a^	109 (69.9%)***	170 (54.5%)	16 (35.6%)	22 (25.6%)	48 (70.6%)	87 (65.4%)	10 (37.0%)	23 (41.1%)
SABA n (%)^a^	98 (62.8%)	192 (61.5%)	19 (42.2%)	48 (55.8%)	36 (52.9%)	61 (45.9%)	7 (25.9%)	11 (19.6%)
SAMA n (%)^a^	10 (6.4%)	20 (6.4%)	1 (2.2%)	2 (2.3%)	12 (17.6%)	22 (16.5%)	0	7 (12.5%)
LABA n (%)^a^	13 (8.3%)	27 (8.7%)	2 (4.4%)	3 (3.5%)	9 (13.2%)*	34 (25.6%)	2 (7.4%)	8 (14.3%)
LAMA n (%)^a^	15 (9.6%)	20 (6.4%)	2 (4.4%)	3 (3.5%)	35 (51.5%)	67 (50.4%)	13 (48.1%)	22 (39.3%)
LABA/ICS n (%)^a^	73 (46.8%)*	114 (36.5%)	16 (35.6%)	28 (32.6%)	27 (39.7%)	55 (41.4%)	3 (11.1%)	16 (28.6%)
LAMA/ICS n (%)^a^	6 (3.8%)	6 (1.9%)	1 (2.2%)	0	13 (19.1%)	27 (20.3%)	1 (3.7%)	1 (1.8%)
ICS n (%)^a^	49 (31.4%)	108 (34.6%)	11 (24.4%)	22 (25.6%)	9 (13.2%)	33 (24.8%)	3 (11.1%)	6 (10.7%)

*ACQ* asthma control questionnaire, *CCQ* COPD control questionnaire, *ICS* inhalation corticosteroid, *GOLD* Global initiative for chronic Obstructive Lung Disease system, *LABA* long-acting beta agonists, *LABA/ICS* long-acting beta agonist in combination with inhalation corticosteroid, *LAMA* long-acting anticholinergics, *MRC* dyspnoea scale Medical Research Counsel dyspnoea scale, *NA* not applicable, *SABA* short-acting beta agonist, *SAMA* short-acting anticholinergics.

^*^ *p* ≤ 0.05, ** *p* ≤ 0.01, *** *p* ≤ 0.001.

^a^The number of patients who received that type of care at least once within the study period.

^b^Total of all pulmonary medication, excluding prednisone/prednisolone and antibiotics.

In both the asthma and the COPD patient groups, we observed no statistically significant differences between persons with or without intellectual disabilities regarding the overall number of patients being prescribed pulmonary medication, nor regarding almost any specific type of pulmonary medication. However, in asthma patients, LABA/ICS (a long-acting beta agonist combined with an inhalation corticosteroid) was being prescribed to significantly more asthma patients with intellectual disabilities than without intellectual disabilities during the study period (46.8% vs 36.5%; p = 0.03). In addition, a significantly larger portion of asthma patients with intellectual disabilities (69.9% vs. 54.5% in the asthma control group) received oral antibiotics prescriptions, prescribed for a broad range of medical conditions (p = 0.001).

In COPD patients, LABA was prescribed to a significantly lower number of patients with intellectual disabilities (13.2%) compared with patients without intellectual disabilities (25.6%; p = 0.04). There were no other statistically significant differences between these groups regarding antibiotics prescriptions.

## Discussion

In a retrospective cohort study, we assessed the year point prevalence of asthma and COPD and compared the asthma- and COPD-related GP care activities between patients with and without intellectual disabilities to investigate potential differences in care delivery and treatment-related patient characteristics in the context of primary care management of asthma and COPD. Compared with matched patients without intellectual disabilities, more asthma patients with intellectual disabilities were prescribed antibiotics. They were also more often obese and/or diagnosed with diabetes, anxiety, or depression. Both asthma and COPD patients with intellectual disabilities were nearly twice as often current smokers and, before matching, appeared considerably younger in age than patients without intellectual disabilities but with asthma or COPD. In the study period, COPD patients with intellectual disabilities, compared with matched patients without intellectual disabilities, received more often either no COPD-related practice consultation at all or a large number of practice consultations. In addition, the asthma prevalence was much higher in patients with intellectual disabilities during the last 6 years, with a nearly 9% point prevalence and almost 3%-point difference in 2019. COPD prevalence was similar for COPD patients with and without intellectual disabilities in 2019.

The increased asthma prevalence we found in adults with intellectual disabilities is consistent with previous studies^12,15,16,18,31^, as is our finding that the COPD prevalence in adults with intellectual disabilities is comparable with that in adults without intellectual disabilities^19^. The relative overrepresentation of young adults with intellectual disabilities and asthma (age 18–34 and age 35–49), however, has not yet been described in the literature, although higher asthma prevalence among *children* with intellectual disabilities has been mentioned in other studies^32,33^. Our finding that COPD patients with intellectual disabilities were considerably younger than their counterparts without intellectual disabilities, resembles a recent study calculating an age- and sex-adjusted prevalence risk for COPD of 1.5 for people with intellectual disabilities^34^.

We found higher rates of current smoking (both asthma group and COPD group) and obesity (asthma group) for patients with intellectual disabilities. For asthma, this has been confirmed in other studies as well^12^, but we found no comparison literature for COPD patients with intellectual disabilities. The overall smoking rates in people with mild intellectual disabilities are comparable to the general population. However, research shows that health-promoting activities including recording smoking status and offering smoking cessation are less likely to be addressed for adult patients with intellectual disabilities^15,35^. Both obesity and smoking are associated with poorer control and/or severity of asthma and COPD^36–38^. Weight management and getting people to quit smoking, which are both aims in the management of asthma and COPD, may apparently pose a challenge for GPs in patients with intellectual disabilities. However, from our study, no general conclusions can be drawn with regard to the quality of the primary care management of asthma and COPD for this patient group. For example, our finding about a relatively high number of asthma patients with intellectual disabilities receiving antibiotics may be an indication of poor asthma management^39^, but our database was not sufficiently detailed to explore the particular conditions for which these antibiotics were prescribed. The Dutch GP guidelines ‘Adult asthma’ and ‘COPD’ do not list intellectual disability, or Down syndrome, as a reason to give antibiotics more easily^40^. Additionally, the number of patients receiving a spirometry assessment did not differ statistically significantly between asthma or COPD patients with and without intellectual disabilities, despite the known difficulties in obtaining accurate spirometry results in people with intellectual disabilities^9,15,41^. In our study, we know that spirometry assessment was initiated in patients with intellectual disabilities. However, we had no access to the results of these assessments, and problems in the actual performance of spirometry may still have occurred.

We observed a higher rate of COPD patients with intellectual disabilities either receiving no COPD-related practice consultation at all or a large number of consultations for COPD. In primary care, people with intellectual disabilities in general have higher consultation rates than GP patients without intellectual disabilities. This has been directly linked to problems in disease management^5^ and higher levels of comorbidity^17,42^. However, contrary to other research^34^, we did not find more comorbidities in COPD patients with intellectual disabilities compared with COPD patients without intellectual disabilities. In addition, higher consultation rates need not necessarily be an indicator for problems in the uptake of disease management; they may also point to good disease control activity. For COPD for example, recent research shows that enrolment of people with intellectual disabilities in primary care COPD disease management programmes is comparable to enrolment of controls without intellectual disabilities, and that enroled patients with intellectual disabilities are more likely than their controls to be frequent consulters (odds ratio 3.01). However, there appeared to be no difference between patients with intellectual disabilities and controls in receiving high numbers of prescriptions of inhalation medication per year (≥2), an indication of poor disease control, or in discussing smoking behaviour. In the group of patients with COPD who were not enroled, those with intellectual disabilities were more likely to receive >2 prescriptions of inhalation medication prescriptions than controls not enroled without intellectual disabilities (odds ratio 1.84)^43^. Enrolment in management programmes may therefore, for people with intellectual disabilities in particular, be an important way of controlling medication use and implementation of other interventions. However, compared with controls without intellectual disabilities, we also found a higher rate of COPD patients with intellectual disabilities receiving no COPD-related practice consultation during our study period, warranting continuing attention on adequate access to COPD care for this patient group. We found no other literature regarding specific COPD consultation rates in people with intellectual disabilities to compare with.

Lastly, in our study, the year-point prevalence for asthma rose in both patient groups each subsequent year. This also applied to the COPD patient groups, although less profoundly. It is unclear how this may be best explained and whether it indicates, for example, an actually increased incidence of asthma/COPD in this study population, or, e.g. improved healthcare access and diagnostics. The partial revision of the Dutch GP guidelines ‘Adult asthma’ and ‘COPD’ in 2015 may have focused GPs’ attention on the two conditions. In addition, revisions in both guidelines, such as redefining the cut-off point for the diagnosis of airway obstruction and new spirometry reference values, may have contributed to more diagnoses^40^. However, the year prevalence numbers in our study were already rising well before 2015 and, during subgroup analyses performed to scrutinise our data, we did not find statistically significant differences between care delivered in the period before and after 2015. Further, with possibly improved life expectancy and stable incidence of asthma/COPD, prevalence numbers will also increase, although recent Dutch national prevalence numbers show a slight decline in COPD prevalence and a stable asthma prevalence. (https://www.vzinfo.nl/copd/leeftijd-en-geslacht; https://www.vzinfo.nl/astma/leeftijd-en-geslacht). Other factors may also contribute, such as potentially altering characteristics of the patient population in our study, relating to an unbalanced inflow and outflow of type and number of patients over the years in the selected practices.

This is one of the few studies investigating both the quantity and the quality of GP-care-related care activities for asthma and COPD for adults with intellectual disabilities, and as such reduces the disturbing scarcity of research on this topic. We used high-quality data from a large primary care dataset with follow-up data in the Netherlands, with a great variation in general practices and representative of the whole Dutch population. Access to detailed GP information on episode titles gave us the opportunity to apply strict inclusion and exclusion criteria, contributing to a refined study population. We were, however, not able to relate consultations specifically to respiratory conditions for all included general practices. Also, levels of intellectual disability could not be identified in the GP medical records. In addition, as primary healthcare access and adequate identification of chronic conditions in people with intellectual disabilities are known to be very challenging^15^, it should be noted that our data relate to patients who did manage to find their way to a GP and where GPs did manage to state a diagnosis. Our prevalence results should therefore be regarded as conservative estimates and may well be underestimates of the actual primary care practice situation.

In this ten-year retrospective primary care cohort study, we found a significantly higher primary care prevalence of asthma and concomitant use of antibiotics among adult patients with intellectual disabilities, compared with asthma patients without intellectual disabilities. Asthma patients with intellectual disabilities also appeared more often obese and diagnosed with diabetes, anxiety, or depression. In addition, both asthma and COPD patients with intellectual disabilities were more often smokers and considerably younger in age than their counterparts without intellectual disabilities. COPD patients with intellectual disabilities received more often either no COPD-related practice consultation at all or a large number of practice consultations. From our study, no general conclusions can be drawn with regard to the quality of the primary care management of asthma and COPD for this patient group. However, our results warrant further research into the causes of the differences found and whether they also infer differences in the quality or the effectiveness of asthma and COPD care for people with intellectual disabilities, especially for young adults with intellectual disabilities.

### Supplementary information

Supplementary Information

## Data Availability

The data that support the findings of this study are available from the corresponding author upon request.
