# Supplementary material for: Asthma and COPD management of patients with intellectual disabilities in general practice
Source: NPJ Prim Care Respir Med. 2024 Jun 26;34:15. doi: 10.1038/s41533-024-00375-w (PMC11208168; doi:10.1038/s41533-024-00375-w)
Supplement: Supplementary file 1 — Supplementary Information [file 41533_2024_375_MOESM1_ESM.docx]

**Supplementary methods**

**Detailed information on the identification of adults with intellectual disabilities**

| **Two-step identification of adults with intellectual disabilities** |
| --- |
| - Selection of adults: - With ICPC code P85: mental retardation/intellectual disabilities AND/OR with ICPC code A90: multiple birth defects such as Down syndrome (A90.01) AND/OR - Adults with ICPC-P and ICPC-A codes relating to intellectual disabilities in combination with episode of care diagnostic labels containing: ‘limit*’, ‘intelligent*’, ‘IQ’, ‘mild ID’, ‘ID’, ‘retard*’, ‘mind’, ‘mentally retarded’, ‘(moderately) retarded’ - Manual investigation of all accompanying episode of care diagnostic labels and exclusion of adults with the following conditions: - Wrongly allocated P85 code, based on the episode of care diagnostic label - Birth defects (code A90) without (explicit mention of) intellectual disabilities as a feature, e.g., tuberous sclerosis, Turner syndrome, Klinefelter syndrome, unspecified chromosomal abnormalities, or unspecified multiple birth defects - Motor retardation or unspecified disability - Cognitive limitations due to other causes, e.g., Alzheimer’s disease, stroke, brain tumour, or acquired brain injury - Mental disorders (code P99) with explicit mention of normal intelligence, or without further information on the level of functioning - An episode of care diagnostic label referring to familial conditions |

ICPC: International Classification of Primary Care; ID: intellectual disabilities/intellectual disability

**Detailed information on the operationalisation and measures of the health record parameters used**

| **Health record parameter** | **Operationalisation and measures** |
| --- | --- |
| Consultation patterns | The total number of GP contacts, for all medical conditions combined, including:   - face-to-face consultations - telephone consultations - home visits during office hours - general practice out-of-hours contacts   It was not possible to distinguish consultations exclusively for respiratory problems.  Measures per type of consultation and for all types combined:   - Number of patients receiving at least one consultation within the research period - Number of consultations per patient |
| Spirometry measurement | Spirometry assessment was considered present based on the presence of at least one of the following FEV1-related measures^1^:   - FEV1 pre-BD - FEV1 pre-BD % of predicted value - FEV1 post-BD - FEV1 post-BD % of predicted value - FEV1/FVC ratio pre-BD - FEV1/FVC ratio post-BD   Measure:   - Number of patients receiving spirometry assessment at least once during the research period |
| Health questionnaires  ACQ, CCQ, MRC dyspnoea | If the results of each of the questionnaires were registered more than once, the most recent registration was used for calculation.  ACQ-6 and ACQ-7 ^2^: contains, respectively, six or seven questions of which five about asthma symptoms and one question about b2-agonist use occurring in the previous seven days, rated by the patient on a scale from 0 to 6. The ACQ-7 contains another question with the same scale about predicted FEV1%, which is completed by the clinic staff. The items are equally weighted. The ACQ score is the mean of the seven items, categorised as: <0.75 good control; 0.75–1.50 moderate control; >1.50 poor control.  Measures:   - The number of patients with a registration of at least one ACQ score - The number of patients with a mean ACQ score of <0.75   CCQ ^3^: contains 10 health-related quality of life questions covering the domains ‘symptoms’, ‘functional state’, and ‘mental state’, rated by the patient based on experiences in the previous seven days on a scale from 0 to 6. The items are equally weighted. The total CCQ score is the mean of the 10 items and ranges from score 0, indicating very good control, to score 6, indicating extremely poor control.  Measures:   - The number of patients with a registration of at least one CCQ score - The median CCQ score within the group of COPD patients with and without ID   MRC dyspnoea ^4^: contains five statements based on perceived breathlessness classified into six severity grades:   - 0, ‘no complaints’ - 1, ‘I only get breathless with strenuous exercise’ - 2, ‘I get short of breath when hurrying on the level or up a slight hill’ - 3, ‘I walk slower than people of the same age on the level because of breathlessness or have to stop for breath when walking at my own pace on the level’ - 4, ‘I stop for breath after walking 100 metres or after a few minutes on the level’ - 5, ‘I am too breathless to leave the house’   In this study, three subgroups of MRC dyspnoea outcomes were created:   - Grades 0–1 - Grades 2–3 - Grades 4–5   Measures:   - The number of patients with a registration of at least one MRC dyspnoea score - The number of patients scoring, respectively, Grades 0–1, Grades 2–3, and Grades 4–5 |
| GOLD severity stage | The GOLD severity stages of COPD – Mild (1), Moderate (2), Severe (3), and Very severe (4) – were rated by the GPs according to the reports of the Global initiative for chronic Obstructive Lung Disease between 2001 and 2019 ^5^. For this study, GOLD stage ratings were grouped into three categories:   - Mild - Moderate - Severe and very severe   Measure:   - The number of patients with, respectively, GOLD stage ‘mild’, stage ‘moderate’, and stage ‘(very) severe’ |
| Pulmonology referrals | Measure:   - The number of patients with at least one referral with ICPC code R95 or R96 to a pulmonologist |
| ATC codes | Codes used for selection of respiratory medication, use of systemic antibiotics, and systemic prednisone/prednisolone ^6^:  R03AC0: short-acting beta agonist (SABA)  R03AC1: long-acting beta agonists (LABA)  R03AK: long-acting beta agonist + inhalation corticosteroid (LABA/ICS)  R03BA: inhalation corticosteroid (ICS)  R03BB01: short-acting anticholinergics (SAMA)  R03BB04-07: long-acting anticholinergics (LAMA)  R03AL: long-acting anticholinergics + inhalation corticosteroid (LAMA/ICS)  J01: antibiotics  H02AB06: prednisolone  H02AB07: prednisone  Measures:   - The number of patients with at least one prescription for each of the abovementioned ATC codes - The number of patients with at least one prescription within the total group of abovementioned R03 codes |
| Comorbidity | The following ICPC codes were used for comorbidity:   - Hypertension: K86, K87 - Ischaemic heart disease: K74, K75, K76 - Heart failure: K77 - Cerebrovascular accident: K90 - Diabetes: T90 - Anxiety/depression: P74, P76 - Osteoporosis: L95 - Osteoarthritis: L84, L89, L90, L91 - Obesity: T83 AND/OR BMI ≥30 |

ACQ: Asthma Control Questionnaire; ATC: Anatomical Therapeutic Classification; BD: bronchodilatation; BMI: Body Mass Index; CCQ: Clinical COPD Questionnaire; FEV-1: forced expiratory volume in 1 second; FVC: forced vital capacity; ICPC: International Classification of Primary Care

**References**

1. Dutch College of General Practitioners (NHG) guideline COPD (M26). Utrecht: Nederlands Huisartsen Genootschap (NHG); 2021. https://richtlijnen.nhg.org/files/pdf/102_COPD_april-2021.pdf

2. Juniper, E., O'Byrne, P., Guyatt, G., Ferrie, P. & King, D. Development and validation of a questionnaire to measure asthma control. *Eur Respir J.* **14**, 902-907 (1999).

3. van der Molen, T. *et al.* Development, validity and responsiveness of the Clinical COPD Questionnaire. *Health Qual Life Outcomes* **1**, 13, doi:10.1186/1477-7525-1-13 (2003).

4. Bestall, J. C. *et al*., Usefulness of the Medical Research Council (MRC) dyspnoea scale as a measure of disability in patients with chronic obstructive pulmonary disease. *Thorax.* **54,** 581-6 (1999).

5. Global Initiative for Chronic Obstructive Lung Disease. *2022 GOLD reports.* https://goldcopd.org/2022-gold-reports/ (2022).

6. World Health Organization Collaborating Centre for Drug Statistics Methodology. *ATC/DDD Index 2022.* https://www.whocc.no/atc_ddd_index/ (2022).
